# Reverse Phenotyping after Whole-Exome Sequencing in Children with Developmental Delay/Intellectual Disability—An Exception or a Necessity?

**DOI:** 10.3390/genes15060789

**Published:** 2024-06-15

**Authors:** Nikola Ilic, Nina Maric, Ales Maver, Lluis Armengol, Ruzica Kravljanac, Jana Cirkovic, Jovana Krstic, Danijela Radivojevic, Sanja Cirkovic, Slavica Ostojic, Stasa Krasic, Aleksandra Paripovic, Vladislav Vukomanovic, Borut Peterlin, Gorica Maric, Adrijan Sarajlija

**Affiliations:** 1 Clinical Genetics Outpatient Clinic, Mother and Child Health Care Institute of Serbia “Dr Vukan Cupic”, 11070 Belgrade, Serbia; ilicnikola91@gmail.com (N.I.); jana.cirkovic@gmail.com (J.C.); jovanakrst98@gmail.com (J.K.); 2 Clinic for Children Diseases, University Clinical Center of the Republic of Srpska, Banja Luka 78000, Bosnia and Herzegovina; ninamaric.bl@gmail.com; 3 Clinical Institute of Genomic Medicine, University Medical Centre Ljubljana, 1000 Ljubljana, Slovenia; ales.maver@kclj.si (A.M.); borut.peterlin@guest.arnes.si (B.P.); 4 CIBER en Epidemiología y Salud Pública (CIBERESP), Genes and Disease Program, Center for Genomic Regulation (CRG-UPF), 08003 Barcelona, Spain; lluis.armengol@qgenomics.com; 5 Department of Neurology, Mother and Child Health Care Institute of Serbia “Dr Vukan Cupic”, 11070 Belgrade, Serbia; ruzicakravljanjac@gmail.com (R.K.); ostojic.slavica@gmail.com (S.O.); 6 Department of Pediatrics, Faculty of Medicine, University of Belgrade, 11000 Belgrade, Serbia; liki_74@yahoo.com (A.P.); vvladavuk64@gmail.com (V.V.); 7 Laboratory of Medical Genetics, Mother and Child Health Care Institute of Serbia “Dr Vukan Cupic”, 11070 Belgrade, Serbia; daca72radivojevic@gmail.com (D.R.); sanja.s.cirkovic@gmail.com (S.C.); 8 Department of Cardiology, Mother and Child Health Care Institute of Serbia “Dr Vukan Cupic”, 11070 Belgrade, Serbia; stasakrasic5@gmail.com; 9 Department of Nephrology, Mother and Child Health Care Institute of Serbia “Dr Vukan Cupic”, 11070 Belgrade, Serbia; 10 Institute of Epidemiology, Faculty of Medicine, University of Belgrade, 11000 Belgrade, Serbia; goricamaric87@gmail.com

**Keywords:** developmental delay (DD), intellectual disability (ID), whole-exome sequencing (WES), reverse phenotyping (RP), neural network analysis

## Abstract

This study delves into the diagnostic yield of whole-exome sequencing (WES) in pediatric patients presenting with developmental delay/intellectual disability (DD/ID), while also exploring the utility of Reverse Phenotyping (RP) in refining diagnoses. A cohort of 100 pediatric patients underwent WES, yielding a diagnosis in 66% of cases. Notably, RP played a significant role in cases with negative prior genetic testing, underscoring its significance in complex diagnostic scenarios. The study revealed a spectrum of genetic conditions contributing to DD/ID, illustrating the heterogeneity of etiological factors. Despite challenges, WES demonstrated effectiveness, particularly in cases with metabolic abnormalities. Reverse phenotyping was indicated in half of the patients with positive WES findings. Neural network models exhibited moderate-to-exceptional predictive abilities for aiding in patient selection for WES and RP. These findings emphasize the importance of employing comprehensive genetic approaches and RP in unraveling the genetic underpinnings of DD/ID, thereby facilitating personalized management and genetic counseling for affected individuals and families. This research contributes insights into the genetic landscape of DD/ID, enhancing our understanding and guiding clinical practice in this particular field of clinical genetics.

## 1. Introduction

Developmental delay (DD) refers to a divergence in the attainment of developmental milestones in children under the age of five years, indicating a lag in one or more developmental domains when compared to peers in the general population [1]. Intellectual Disability (ID), previously referred to as “mental retardation”, denotes inadequate mental development in individuals aged five years and above, resulting in notable restrictions in overall mental capabilities, intellectual functioning, adaptive behavior, and functional skills [2]. These conditions may manifest as an isolated trait in non-syndromic ID (NSID) or in conjunction with a variety of other anomalies, being termed then as a syndromic ID (SID) [3].

The estimates suggest that DD and ID affect 1–3% of the global population, with a male-to-female ratio of 1.6:1 [4,5]. Various genetic and environmental factors can influence the occurrence of DD and ID [4]. The genetic basis of DD and ID is profoundly heterogeneous, involving more than 2000 genes in various pathways and biological processes regulating synaptic activity, chromatin remodeling, gene transcription, protein degradation, and many others [6]. Potentially preventable causes acting during intrauterine development include maternal malnutrition and nutrient deficiencies, prenatal infections, maternal exposure to toxic substances, prematurity, and perinatal asphyxia [7]. 

For children with DD and ID, a systematic diagnostic approach is crucial to uncover the specific cause. A stepwise clinical evaluation is fundamental in the assessment of developmental disorders [8,9]. Despite extensive and often time-consuming and expensive testing, up to 50% of children may not achieve a definitive genetic diagnosis [10]. Chromosomal microarray analysis (CMA) is recommended as the primary genetic diagnostic test for undiagnosed DD and ID cases [11]. However, the capability of detecting pathogenic variants has led to the widespread use of Next-Generation Sequencing (NGS) techniques like targeted gene panels (TGP), clinical exome sequencing (CES), and whole-exome sequencing (WES) in these patients [12]. 

Currently, WES finds its greatest application in diagnosing rare, unclear clinical conditions with atypical or highly nonspecific manifestations, where other diagnostic methods have failed to identify a clear cause of the disease. This method examines the totality of human exome-revealing genetic variations underlying various conditions, being particularly valuable for diagnosing nonspecific disorders with presumed genetic causes, such as DD and ID, congenital malformations, and multisystemic diseases, as well as different monosystemic disorders (e.g., cardiomyopathies, epileptic syndromes, or idiopathic hepatic diseases, etc.) [13]. Genetic findings often reveal variants associated with ultra-rare conditions, unforeseen during clinical investigation of the patient. This scenario prompts the need for a clinical reevaluation of specific clinical features in relation to the diagnosis proposed by the genetic findings. The procedure of reevaluating the patient in the context of genetic findings is designated as reverse phenotyping (RP) [14]. Several studies have shown that RP increases the yield of genetic diagnostic testing, including WES [15,16,17]. 

The primary objective of our study is to estimate the need for RP in patients with DD or ID after receiving positive WES findings. A secondary aim was to reevaluate the efficacy of WES as a diagnostic tool in clinical practice in order to refine its optimal place in the sequence of genetic tests indicated in DD and ID patients.

## 2. Materials and Methods

### 2.1. Study Design and Settings

Our research was methodologically framed as the retrospective descriptive research design. During the period from March 2015 to March 2022, a total number of 100 pediatric patients with DD/ID were recruited for this study at two university hospitals. The study was approved by the Institutional Ethics Committees (numbers: 8/122 and 01-5298/24). All patients were evaluated by clinical geneticists before indicating WES. WES was performed on 100 pediatric patients with DD or ID in the Clinical Institute of Medical Genetics at University Medical Centre Ljubljana, Slovenia (56 patients) and Quantitative Genomic Medicine Laboratories, Barcelona, Catalonia, Spain (44 patients). The same demographic and clinical parameters were collected and used in the statistical analysis for the entire study population using the same methodology: the age of the patient at the time of genetic testing, sex, the presence of facial dysmorphism, the presence of skeletal deformities, the presence of epilepsy, cardiac, renal, endocrine, and/or hepatic clinical abnormality, impairment of vision or hearing, metabolic abnormalities (such as metabolic acidosis, hyperammonemia, hypoglycemia, abnormal amino acid or urinary acid profile, elevated urinary glycosaminoglycans), history of significant perinatal complications, and finally the result of whole-exome sequencing with emphasis on the inheritance mode in case of an established diagnosis. Informed consent for molecular diagnostics and use in further research was obtained from the parents. 

### 2.2. Data Curation 

The demographic, clinical, and genetic data utilized in this study were obtained from medical records, encompassing both paper and electronic formats, sourced from two clinical institutions: The Mother and Child Health Care Institute of Serbia “Dr Vukan Cupic” in Belgrade, Serbia, and the Clinic for Children Diseases, University Clinical Center of the Republic of Srpska in Banja Luka, Bosnia and Herzegovina. The data collection period spanned from March 2015 to March 2022. The dataset comprises 100 records, encompassing various features such as patient age, gender, diagnosis, treatment details, and outcomes, including reports of WES provided by the aforementioned genetic laboratories.

Data cleaning involved removing duplicate entries and correcting any obvious data entry errors. We conducted a thorough review of the dataset to ensure completeness and accuracy. No missing data were identified during this process. Patient records were selected based on specific criteria, including age (6 months to 18 years), gender (both female and male), and diagnosis of DD/ID as per standardized assessment tools. Exclusion criteria were established to ensure sample homogeneity and minimize confounding factors. Thus, we excluded patients with previously incomplete medical records, those younger than 6 months, and in cases of non-standardized developmental assessment.

### 2.3. Measurements

In addition to WES conducted for all patients in the cohort, other genetic analyses had previously been performed in the majority of the patients, but not in a uniform manner, and they included one or more of the following tests: karyotype analysis, multiplex ligation-dependent probe amplification (MLPA) for microdeletion/microduplication syndromes (SALSA MLPA Probemix P064 Microdeletion Syndromes-1B, MRC Holland), chromosomal microarray and molecular analysis for fragile X syndrome (AmplideX PCR/CE FMR1 kit, Asuragene USA). Metabolic investigations were performed in case of suspicion of inherited metabolic disorder and included blood lactate, ammonia, amino acids profile, and urinary metabolic screen (amino acids, organic acids, glycosaminoglycans).

Exome sequencing in Quantitative Genomic Medicine Laboratory in Barcelona was performed using two distinct probe sets: MedExome and HyperExome. The MedExome probes targeted 46.6 Mbp of the genome and focused on databases including CCDS 17, RefSeq, Ensembl 76, VEGA 56, GENCODE 20, and miRBase 21. The HyperExome probes targeted 42.8 Mbp of the genome and covered CCDS release 20, RefSeq (30 July 2018), Ensembl release 93, and GENCODE release 28. Exome sequencing at Clinical Institute of Genomic Medicine in Ljubljana utilized either the Illumina Nextera Rapid Capture Exome or the Twist Core Exome. The Illumina Nextera Rapid Capture Exome covered 37 Mbp of the genome, while the Twist Core Exome covered 33 Mbp of the genome. Although mitochondrial sequences were not included in the capture during the exome-sequencing process, off-target reads were analyzed to evaluate mitochondrial DNA. This analysis was performed using specific bioinformatics tools designed to identify and quantify mitochondrial sequences from off-target reads.

Sequencing was initially performed in the proband, and in the case of detection of a potentially causative variant, subsequent validation and segregation analysis using Sanger sequencing was performed on parental samples. Pathogenicity of the detected variants was classified according to the American College of Medical Genetics and Genomics and the Association for Molecular Pathology guidelines [18]. 

Reverse phenotyping (RP) was applied in patients with positive whole-exome sequencing (WES) findings in unforeseen genes. The approach involved scrutinizing the clinical presentations of these patients alongside their genetic data to uncover genotype–phenotype correlations. Therefore, RP encompassed comprehensive clinical reevaluation coupled with additional investigations tailored to the likely diagnosis unveiled by WES results. This methodology aimed to identify distinctive features associated with specific genetic variants, thereby refining final diagnoses.

### 2.4. Statistical Analysis

Statistical analysis included descriptive and analytical methods. Pearson chi-square and Fisher exact tests were used for frequency comparison between the groups. Logistic regression was employed to establish factors associated with a high probability of positive results of WES and subsequent need for RP. Results are presented as count (%). All *p*-values lower than 0.05 were considered significant. Data were analyzed using SPSS, version 20.0 (IBM Corp., Armonk, NY, USA).

This study utilized neural network analysis by means of SPSS 20 to investigate predictive factors associated with positive WES results in a cohort of 100 patients diagnosed with DD/ID. Input factors included fourteen characteristics (all clinical parameters, age, and sex). The hidden layer comprised 8 units with a hyperbolic tangent activation function. The output layer consisted of two units representing diagnosis, utilizing SoftMax activation and cross-entropy error function. The neural network model was trained on a subset of the data to identify patterns and relationships between predictor variables and the likelihood of a positive WES result. Subsequently, the model was evaluated using a testing dataset to assess its predictive accuracy and generalization capability. The same methodology was used for performing neural network analysis to investigate predictive factors associated with the necessity of RP in patients with WES findings.

## 3. Results

A total of 100 patients, 59 males and 41 females, were included in the study. The age of the studied population ranged from 1 month to 16.5 years with a median age of 28 months. Key clinical manifestations and organ involvements in patients with confirmed genetic causes of DD/ID are presented in Table 1. 

The most commonly performed genetic analysis before WES was MLPA for microdeletions and microduplications (46 cases) while chromosomal microarray was conducted in 7 cases. All previous findings were negative. The diagnostic yield of WES was not significantly different between the group of patients with previous genetic analyses and those without pre-WES analyses (chi-square = 1.931, *p* = 0.381). However, the need for RP was significantly higher in patients with previously negative genetic testing when compared to patients lacking pre-WES analysis (chi-square 8.737, *p* = 0.013). 

Genetic diagnosis was established by means of WES in 66 patients (66%). The detected variants and associated conditions are presented in Supplementary Table S1. The consistency of the positive findings between the two laboratories involved in this study was exceptionally uniform: the proportion of positive results was 66,1% (37/56) in the Clinical Institute of Medical Genetics at the University Medical Centre Ljubljana, while in the Quantitative Genomic Medicine Laboratories in Barcelona, positive findings were reported in 65.9% patients (29/44) (chi-square value 1.0 exact two-sided significance at 0). 

We report 60 unique affected genes associated with distinct genetic conditions (including deletion syndromes involving multiple genes). All patients had unique causative genetic variants, as shown in Supplementary Table S1. The most frequent gene affected is *PDHA1*, found in three patients, followed by *MECP2*, *TCF4*, *CHD3*, and *KMT2D*, each repeated twice in our cohort. The most common inheritance mode of the disorders diagnosed was autosomal dominant (45%), followed by X-linked (12%), autosomal recessive (8%), and mitochondrial (1%). 

Logistic regression was employed to ascertain factors affecting the probability of establishing a diagnosis by WES in pediatric patients with DD/ID. Demographic factors (age, gender) and clinical parameters did not significantly affect the result of the WES when evaluated by logistic regression. However, the presence of epilepsy negatively affected the likelihood of establishing a diagnosis by WES, with borderline significance (*p* = 0.088). Additionally, eight out of nine patients with metabolic abnormalities detected by a standard metabolic screen of blood and urine had causative variants found by WES, but this finding failed to reach the level of statistical significance (chi-square = 2.309, *p* = 0.161). 

There was no statistically significant difference in the mean age of diagnosis between the group of patients diagnosed and undiagnosed using WES (24 vs. 38 months, respectively, Mann–Whitney U = 874, *p* = 0.071).

Reverse phenotyping was indicated in 33 patients after receiving results of WES (33% of the total number, and half of the patients with positive WES findings). There was no significant difference in age between patients with and without indication for the RP (Mann–Whitney U = 453, *p* = 0.24). Clinical parameters that significantly affected the probability of the need for RP in children with DD/ID after WES (when tested with logistic regression) were the presence of metabolic (*p* = 0.003) and renal abnormalities (*p* = 0.024). Renal abnormalities were significantly more frequent among patients requiring RP, while metabolic abnormalities were significantly more frequent among cases not requiring RP. This finding was reconfirmed by the chi-square analysis. 

The neural network model used to investigate predictive factors in children with developmental delay/intellectual disability (DD/ID) associated with positive whole-exome sequencing achieved a training cross-entropy error of 25.442 with 18.6% incorrect predictions, while the testing yielded a cross-entropy error of 6.747 with 17.6% incorrect predictions. The overall prediction accuracy was 81.4% for training and 82.4% for testing.

The model’s performance was evaluated using precision, recall, and F1 scores. For the testing sample, the precision and recall for the positive diagnosis were 78.6% and 100%, respectively, resulting in an F1 score of 88%. For the negative diagnosis, the precision was 100% and the recall was 50%, leading to an F1 score of 67%. The macro-averaged F1 score, which considers both classes, was 77.5%, indicating that the model performs well in identifying positive diagnoses but has some limitations in accurately identifying negative diagnoses.

The neural network model used to predict the need for reverse phenotyping in cases of positive whole-exome sequencing (WES) findings achieved a training cross-entropy error of 1.301 with 0% incorrect predictions, while the testing yielded a cross-entropy error of 3.192 with 16.7% incorrect predictions. The overall prediction accuracy was 100% for training and 83.3% for testing, indicating strong predictive ability during training but moderate performance on unseen data.

The model’s performance was evaluated using precision, recall, and F1 scores. For the testing sample, the precision and recall for predicting the need for reverse phenotyping were both 100%, resulting in an F1 score of 100%. For the negative prediction (no need for reverse phenotyping), the precision was 66.7% and the recall was 100%, leading to an F1 score of 80%. The macro-averaged F1 score of 90%, documents good performance in identifying cases needing reverse phenotyping but limited capability of predicting cases not needing reverse phenotyping.

The contribution of each demographic variable to the predictive model, with normalized importance values providing a relative measure within the model, is presented in Table 2.

## 4. Discussion

Developmental delay/intellectual disability (DD/ID) represents a group of genetic, phenotypic, and clinically heterogeneous group of disorders that affect approximately 1–3% of children worldwide [1,3]. Timely diagnosis provides the possibility of more accurate clinical management, eventual access to a more effective treatment, coupled with effective genetic counseling to relatives, therefore implicitly providing improvement in the quality of life of the patient and affected families [1]. A systematic diagnostic approach is essential for identifying specific genetic causes in children with DD/ID [19]. The current diagnostic yields suggest the application of WES as a routine first- or second-tier diagnostic tool permitting an early etiological diagnosis for DD/ID patients [20]. 

In our investigation, WES led to the diagnosis in 66% of the pediatric patients with DD/ID. A study in Japan, involving a cohort group like ours produced analogous findings, detecting pathogenic variants in 58 out of 101 patients [21]. A smaller study from South Korea with 41 patients with DD/ID gave a diagnostic yield of WES 48.8% [22]. A Dutch study presented results from 100 consecutive patients with both epilepsy and ID, in which the diagnostic WES analysis reported 1 or more variants providing definitive diagnosis for 58 of them [23]. In a Brazilian study investigating the effectiveness of different diagnostic approaches in pediatric patients with DD/ID, gene panel analysis emerged as the most efficient testing method, successfully identifying mutations in 63.1% (12/19) of cases [24]. In an investigation carried out in Oman, 215 patients afflicted with DD/ID from 118 Middle Eastern families were enrolled. WES was conducted for 188 individuals, revealing pathogenic or likely pathogenic variants in 32 of the 118 families (27%). Variants of uncertain significance were identified in 33 families (28%), while candidate genes potentially linked to ID were detected in 32 families (27%), impacting a total of 64 individuals [25]. In a meta-analysis encompassing 103 studies involving 32,331 individuals with DD/ID and epilepsy, targeted gene panel sequencing was employed in 73 cohorts, and WES was utilized in 36 cohorts. Across these highly selected patient cohorts, the diagnostic yield was 17.1% for DD, 24% for epilepsy, and 28.2% for ID, resulting in an overall yield of 23.7%. Notably, the highest diagnostic yield for epilepsy subtypes was observed in individuals with ID 27.9% and early-onset seizures 36.8% [19]. We speculate that the slightly increased diagnostic yield of WES in our DD/ID cohort compared to similar studies may result from a stringent selection process of patients for receiving WES analysis. Additionally, our results suggest a higher yield of WES when compared to a very similar cohort of patients analyzed by the clinical exome sequencing (CES) method [26]. 

The most commonly performed genetic analysis before WES was MLPA analysis for microdeletions and microduplications (46 cases), while chromosomal microarray was performed in seven cases, and all findings were negative. The diagnostic yield of WES was not significantly different between the group of patients with the aforementioned previous genetic analyses performed and those without pre-WES analyses. This aligns with recent studies suggesting that WES serves as an effective and reliable first-tier diagnostic test in the evaluation of children with DD/ID [20].

Neural network models designed during our study showed reasonable predictive ability for identifying patients with a high probability of positive WES findings and commendable efficacy in the classification of patients needing clinical re-evaluation after receiving positive genetic findings (reverse phenotyping). A similar study evaluating the predictive ability of neural networks in selecting patients with DD/ID for WES demonstrated that testing patients with the top 10% probability of positive WES results in more than double the diagnostic yield of WES, from 26% to 56% [27]. Adding to that, the study highlights that 92% of patients with the lowest prediction scores accurately receive negative WES results, reinforcing the model’s efficacy in patient selection for WES. Despite these promising results, it is important to note that predictive models based on the use of artificial intelligence often lack adherence to standardized reporting specifications, such as those outlined in The Transparent Reporting of a multivariable prediction model for Individual Prognosis Or Diagnosis (TRIPOD) guidelines [28]. This includes inadequate handling of missing values and outliers in the reporting process, as well as a failure to disclose the threshold of the model. Therefore, their use should be mindful, and the results of these models should always be interpreted within the broader context of the clinical decision-making process. Additionally, our finding of overall prediction accuracy for positive WES result of 82.4% on the testing set, demonstrated the model’s capability in generalizing to unseen data. However, despite the high precision and perfect recall for positive diagnoses (resulting in an F1 score of 88%), the model struggled with negative diagnoses, as evidenced by a lower F1 score of 67%. This disparity suggests that while the model is proficient in correctly identifying positive cases, it has a higher rate of false positives for negative cases. We could assume that integrating more diverse predictive factors could further enhance the model’s performance and reliability in clinical applications. Similarly, the neural network model demonstrated robust performance in predicting the need for reverse phenotyping in cases with positive whole-exome sequencing (WES) findings. With perfect prediction accuracy during training and an overall accuracy of 83.3% on the testing set, the model shows the potential for clinical application in identifying cases requiring further phenotypic analysis. The perfect recall for both positive and negative predictions highlights the model’s capability to identify true cases accurately. However, the lower precision for negative predictions indicates a propensity for false positives, suggesting areas for further refinement.

The logistic regression model did not identify any statistically significant predictors of positive WES results (*p* > 0.05 for all variables), with variables such as epilepsy, sex, age, and metabolic conditions showing limited predictive power. In contrast, the neural network model demonstrated robust predictive performance, achieving an F1 score of 0.88. The neural network model’s capability to correctly identify cases with a high probability of positive WES findings (100% recall in the testing set), and its higher overall percent correct predictions (82.4%), highlights its superiority in capturing complex relationships among the same predictive factors used in the logistic regression model.

The contributions of demographic variables to the neural network model, as shown in Table 2, highlight the varying importance of different demographic and clinical factors in predicting positive WES findings and the need for reverse phenotyping, respectively. Notably, age and prior genetic analysis showed the biggest influence on predictions, suggesting that older age and negative previous genetic testing increase the likelihood of positive WES findings. This underscores the necessity of considering these variables in clinical assessments to improve diagnostic accuracy and patient outcomes.

The need for RP notably increased among patients with negative results of other genetic testing before undergoing WES, compared to those without pre-WES analysis. We can speculate that patients with negative genetic testing results (chromosomal microarray, MLPA for microdeletions and microduplications, and others), prior to undergoing WES, are more likely to have a nonspecific clinical presentation and therefore a more challenging diagnostic journey. This could have prompted a higher demand for RP in this subset of patients, indicating a need to revisit clinical and phenotypic features upon receiving positive WES results.

We report 60 unique genes or genetic conditions (including deletion syndromes involving multiple genes). The most frequent gene affected is *PDHA1*, found in three patients, followed by *MECP2*, *TCF4*, *CHD3*, and *KMT2D,* each repeated twice in our cohort. These results exhibit distinctive characteristics compared to previous worldwide studies, which is somewhat expected given the significant heterogeneity in the etiology of DD/ID [19,20,21,22,23,24,25]. 

The most common inheritance mode of the conditions diagnosed in our cohort of patients was autosomal dominant (45%), followed by X-linked (12%), autosomal recessive (8%), and mitochondrial (1%), which is in agreement with previously reported findings [19,20,21,22,23,24,25]. 

Previous research mostly indicates a higher prevalence of DD/ID in males [19,20,21,22,23,24,25]. Our study aligns with this trend, demonstrating a greater representation of male patients with a male-to-female ratio of 1.4:1. The majority of participants in our study fell within the 1 to 3 years range when referred for WES diagnosis, a pattern consistent with previous study findings [19,20,21,22,23,24,25].

Our study revealed a marginally significant age difference between children diagnosed through WES (24 months) and those with negative WES results (38 months). Notably, individuals with a genetically attributed condition exhibited DD at an earlier age, typically within the first year. This observation may contribute to the higher confirmation rate of WES diagnoses in younger patients [25]. 

In 84% of patients from our group, in addition to DD/ID, other symptoms and signs were also present. The most common clinical characteristics were facial dysmorphism, epilepsy, and vision impairment, complementary to similar studies [20,21,22,23,24,25].

The role of epilepsy in determining the likelihood of a diagnosis by WES demonstrated marginal significance in our study. Specifically, the presence of epilepsy was found to have an adverse impact on the probability of establishing a diagnosis via WES. Interestingly, our results diverge from another study, where patients with epilepsy were shown to be 1.5 times more likely to obtain positive diagnostic exome sequencing results compared to individuals without epilepsy [29]. Likely, this divergence is associated with the inclusion criteria and the method of patient recruitment.

A systematic review of 48 previous studies assessing the co-occurrence of epilepsy and ID, revealed an overall prevalence rate of 22.2% [19]. This result agrees with our finding of 25% of patients with epilepsy in our cohort.

In eight out of nine patients exhibiting metabolic abnormalities, WES successfully established an accurate diagnosis, underscoring the method’s substantial yield in the realm of inborn errors of metabolism. Despite the noteworthy outcome, the relatively limited sample size prevented the attainment of statistical significance for this observation. Consistent findings are published in a previous report reviewing existing literature [30], which emphasizes the efficacy of WES in detecting both metabolic and neurologic diseases. Moreover, another study reported a diagnostic success rate of 68% in cases of DD/ID and unexplained metabolic abnormalities when utilizing WES [31].

Although the diagnosis for both patients exhibiting liver abnormalities associated with DD/ID was successfully determined through WES, this outcome did not achieve statistical significance, likely attributed to the relatively small number of patients with affected livers in this cohort. Zellweger syndrome and propionic acidemia were among the diseases identified in our patients with liver pathology. Notably, it was observed that almost one-half of all pediatric patients with chronic liver disorders have an underlying genetic cause, highlighting the potential significance of liver involvement as a predictor for a positive diagnosis [32]. 

Despite the relatively high diagnostic yields of WES in identifying genetic variants associated with suspected genetic disorders, challenges persist in reaching a conclusive diagnosis. One obstacle is the non-identification of pathogenic variants, which may be situated in non-coding regions of the genome, which are not interrogated by the exome sequence [33,34]. Additionally, well-covered regions may harbor variants that are not easily detectable by short-read sequencing, such as repeat polymorphisms, single nucleotide variants, or small insertions/deletion polymorphisms [35]. Another complexity arises when a variant is not recognized as pathogenic. Genomes inherently contain numerous gene-damaging and rare variants absent from databases, and distinguishing between those contributing to disease and those that do not, poses a primary challenge in exome sequencing interpretation [33,35].

Variants not identified in known disease genes may go unnoticed as pathogenic. In instances where a single patient exhibits a damaging mutation in a gene not previously linked to the disease, a diagnosis based solely on that information is unlikely [36]. Leveraging various databases becomes crucial in such cases, helping to ascertain if other patients with similar phenotypes have been reported with variants in the same gene [37]. Notably, reports suggest that re-analysis of exome data from patients with initially negative WES results can enhance diagnostic yields by 10–20% [36,37,38].

Further improvement in diagnostic yields through WES can be achieved by employing RP, a procedure defined as the reassessment of clinical features in light of genetic findings, which involves determining the clinical relevance of previously identified potential genetic causes determined via WES [13,14,15,16]. Methodologically, RP entails studying the complexity of genotype–phenotype associations, drawing evidence from published peer-reviewed literature and publicly available databases. Computational tools like VarformPDB facilitate the compilation of genes and variants related to a disease from databases such as HPO, Orphanet, OMIM, ClinVar, and UniProt, along with PubMed abstracts. Biobanks linking electronic health records (EHRs) and genetic data offer a cost-effective means to enhance clinical characterization, enabling deep clinical phenotyping and multi-omics analyses. Tools such as DECIPHER, Matchmaker Exchange, and VarSome aid in integrating and analyzing genetic and phenotypic data [16].

The true potential of this method is exemplified by a pediatric nephrology study that utilized RP within the context of nephrotic syndrome (NS). A cohort of 111 patients was examined, 64 of them steroid-resistant and 47 steroid-sensitive. By using a personalized diagnostic workflow, RP revealed previously unnoticed clinical indicators, unveiling an unexpected underlying genetic nephropathy in 28% of patients with the drug-resistant form of nephrotic syndrome [15].

Another example is a study of patients with Lofgren’s syndrome. Using the RP procedures, researchers discovered distinct, previously unknown patterns in this syndrome: erythema nodosum, a skin rash, is more common in women, while men are more likely to experience bilateral periarticular arthritis and joint inflammation [39]. In the research on pulmonary arterial hypertension (PAH), RP reveals distinct clinical manifestations and outcomes associated with certain genetic variants, particularly in genes like *BMPR2*, *TBX4*, and *KDR*. It underscores the crucial role of genotype–phenotype correlations in understanding disease heterogeneity and guiding personalized treatment strategies [16]. The application of RP has not only advanced our understanding of known diseases but has also played a pivotal role in uncovering novel medical conditions. For instance, RP has facilitated the discovery of previously unrecognized disorders like Koolen–DeVries syndrome, characterized by a 17q21.31 microdeletion impacting the *KANSL1* gene, as well as Potocki–Lupski Syndrome, attributed to a 17p11.2 microduplication [40,41]. 

Diagnoses achieved by WES and reconfirmed by RP included, among others, 23 ultra-rare diseases among our cohort, some of them diagnosed for the first time in our country. An illustrative example in our study is SSADH deficiency confirmed in a child with the typical neurologic presentation of this disease but with unexpected sensorineural hearing loss (SNHL), which required thorough reverse phenotyping. The international registry for this rare inherited disorder of neurotransmitter metabolism did not include any patients with such an association [42]. We could speculate that in the absence of other variants affecting hearing in this patient, the SNHL in our SSADH deficiency patient contributes to understanding the phenotypic spectrum of this specific disease [43]. On the other hand, few metabolic patients in our study required RP since the referral diagnosis was foreseen. This could be explained by the presence of specific metabolic abnormalities detected in these children by biochemical analysis prior to WES. 

Demographic characteristics in our study cohort did not significantly affect the need for RP. Clinical features with a significant impact on a higher probability of requiring RP after WES in children with DD/ID were renal abnormalities, as indicated by logistic regression (*p* > 0.05). Based on these findings, we may speculate that renal issues may be more common among children whose genetic causes of DD/ID are difficult to identify through standard genetic testing methods like WES. These diagnostic findings also have implications for genetic counseling since parents of children with DD/ID and renal abnormalities may benefit from additional counseling regarding the potential genetic factors underlying their child’s condition and the importance of comprehensive diagnostic approaches like RP.

## 5. Limitations of the Study

This study highlights the diagnostic potential of WES in pediatric patients with DD/ID and the utility of RP in refining diagnoses. However, limitations of the study include the relatively small sample size of 100 patients compared to the frequency of the DD/ID pathology, retrospective design, partially tentative statistical associations, and potential ethnic homogeneity within the study population, necessitating cautious interpretation and further validation. Although the WES captures employed by the two laboratories involved in this study differ in size and specific targeted regions, the capacity to detect target mutations was maintained across the different methods. Therefore, we do not anticipate any bias introduced by the different WES captures used in this study. 

## 6. Conclusions

In conclusion, our study underscores the efficacy of WES as a valuable diagnostic tool in elucidating the genetic underpinnings of DD/ID in pediatric populations. With a diagnostic yield of 66%, WES offers significant potential in identifying causative genetic variants, particularly in cases where previous genetic analyses have been inconclusive. The neural network model’s capability to identify cases with a high probability of positive findings of WES suggests its superiority in recognizing complex relationships among the same predictive factors used in logistic regression.

Our findings highlight the importance of RP in cases with negative results of genetic testing prior to WES, indicating the complexity of these patients. Furthermore, our study emphasizes the heterogeneity of genetic causes associated with DD/ID including a plethora of unforeseen ultra-rare diseases. Moving forward, integrating RP into clinical practice holds promise in enhancing diagnostic accuracy in children with DD/ID and associated comorbidities.

## Figures and Tables

**Table 1:** Clinical features and their frequency in the study group.

Clinical Feature	Frequency (%)
DD/ID ^1^ Facial dysmorphism Epilepsy Visual impairment Perinatal complications Cardiac abnormalities Renal abnormalities Endocrine abnormalities Hearing impairment Metabolic abnormalities Skeletal abnormalities Liver impairment	100 33 25 33 25 22 14 13 13 10 10 9 8 2

^1^ Developmental delay/Intellectual Disability.

**Table 2:** Importance of demographic and clinical variables in predicting positive whole-exome sequencing (WES) findings and the need for reverse phenotyping.

	Independent Variable Importance for the Neural Network Model Employed in Predicting Positive WES Finding		Independent Variable Importance for the Neural Network Model Employed in Predicting Need for Reverse Phenotyping	
	Importance	Normalized Importance	Importance	Normalized Importance
Age	0.217	100.00%	0.164	100.00%
Sex	0.03	13.80%	0.089	54.00%
Genetic analysis performed prior to WES	0.126	58.30%	0.135	82.00%
Epilepsy	0.068	31.40%	0.038	23.40%
Facial dysmorphism	0.072	33.10%	0.061	37.10%
Skeletal dysplasia	0.119	54.80%	0.027	16.30%
Endocrine abnormalities	0.056	25.60%	0.048	29.20%
Heart disease	0.054	24.90%	0.022	13.50%
Renal abnormalities	0.035	15.90%	0.146	88.90%
Metabolic abnormalities	0.063	29.00%	0.131	79.80%
Vision impairment	0.021	9.80%	0.033	20.20%
Hearing impairment	0.083	38.20%	0.063	38.50%
Liver abnormalities	0.024	11.20%	0.03	18.20%
Perinatal complications	0.032	14.50%	0.012	7.30%

## Data Availability

The data presented in this study are only available on request from the corresponding author due to privacy or ethical restrictions.

## Supplementary Materials

The following supporting information can be downloaded at: https://www.mdpi.com/article/10.3390/genes15060789/s1, Table S1: Genetic testing summary.
