# Supplementary material for: Reverse Phenotyping after Whole-Exome Sequencing in Children with Developmental Delay/Intellectual Disability—An Exception or a Necessity?
Source: Genes (Basel). 2024 Jun 15;15(6):789. doi: 10.3390/genes15060789 (PMC11203244; doi:10.3390/genes15060789)
Supplement: Supplementary file 1 [file genes-15-00789-s001.zip › genes-3017928-supplementary.pdf]

Table S1. Key clinical manifestations and genetic findings in patients with confirmed genetic etiology of DD/ID

Case	Sex	Phenotype	Gene	Variant	Inheritance	Variant type/ACMG classification	OMIM
**1.**	M	DD, SD, cataract, strabismus, myopia	*LONP1*	NM_004793.4 c.2014C>T p.Arg672Cys	AR	Missense/pathogenic variant	CODAS syndrome OMIM:600373
**2.**	M	DD, LA, encephalopathic crises	*PDHA1*	NM_001173456 c.C522G p.F174L	X	Missense/pathogenic variant	Pyruvate dehydrogenase E1-alpha deficiency OMIM:312170
**3.**	F	DD, encephalopathic crises	*PCCA*	NM_001127692 c.839dupT p.I280fs	AR	Insertion/likely pathogenic	Propionic acidemia OMIM: 606054
				NM_000282.4 c.T1964G p.V655G		Missense/ unknown significance	
**4.**	F	ID, epilepsy	*CACNA1D*	NM_001128840.3 c.C2621A p.P874Q	AD	Missense/unknown significance	Primary aldosteronism, seizures, and neurologic abnormalities OMIM: 615474
**5.**	F	ID, Epilepsy, SM	*PIK3R2*	NM_005027 c.2183G>A p.Arg728His	AD	Missense/pathogenic	Megalencephaly-polymicrogyria-polydactyly-hydrocephalus syndrome 1 OMIM: 603387

**6.**	M	DD, sensoneural hearing loss	*GJB2*	NM_004004 c.71G>A p.W24X	AR	Nonsense/pathogenic	Deafness, autosomal recessive 1A OMIM: 220290
			KAT6A	NM_006766 c.2904G>C p.R968S	AD	Missense/ unknown significance	Arboleda-Tham syndrome OMIM:616268
**7.**	M	Noonan syndrome susp.	*PTPN11*	NM_002834 c.417G>C p.Glu139Asp	AD	Missense/pathogenic	Leopard syndrome OMIM:151100
**8.**	M	DD, hypotonia, FD	*ARID1B*	NM_001244813 c.4774G>T p.Gln84Ter	AD	Missense/likely pathogenic	Coffin-Siris syndrome 1 OMIM: 135900
**9.**	M	DD, SD	*MYH3*	NM_002470 c.533+1G>A	AD	Splicing/likely pathogenic	Arthrogryposis, distal, type 2A (Freeman-Sheldon) OMIM:193700
			SYNGAP1	NM_006772 c.C892T p.P298S		Missense/ unknown significance	Intellectual developmental disorder, autosomal dominant 5 OMIM:612621
**10.**	F	DD, LA, epilepsy, FTT	*SCN2A*	NM_001040143 c.2567G>A p.Arg856Gln	AD	Missense/ unknown significance	Developmental and epileptic encephalopathy 11 OMIM:613721
**11.**	F	ID, FD, hyperactivity, SM	*TCF4*	NM_001083962.2 c.771C>G p.Asp257Glu	AD	Missense/ unknown significance	Pitt-Hopkins syndrome OMIM: 610954
**12.**	F	ID, FD	*DDX3X*	NM_001193417 c.1641insT	X	frameshift/ pathogenic	Intellectual developmental disorder, X-linked syndromic, Snijders Blok type

							OMIM: 300958
**13.**	F	ID, SM	*NALCN*	NM_052867<br>c.2003_2006del	AD	Frameshift/ pathogenic	Hypotonia, infantile, with psychomotor retardation and characteristic facies 1<br>OMIM: 615419
**14.**	M	DD, myoclonic epilepsy, unilateral renal agenesis, FD	*GATA3*	NM_001002295<br>c.G827a<br>p.R276q	AD	Missense/pathogenic	Hypoparathyroidism, sensorineural deafness, and renal dysplasia – Barakat syndrome<br>OMIM: 146255
			NSD1	NM_022455<br>c.A3570t<br>p.L1190f		Missense/ unknown significance	Sotos/Beckwith_Wiedemann sy<br>OMIM :130650
**15.**	F	ID, SD	*RPS6KA3*	NM_004586.3<br>c.1603-1G>A	X	Missense/ unknown significance	Coffin-Lowry syndrome<br>OMIM: 303600
**16.**	M	DD	*TCF4*	NM_001243226.2<br>c.826C>T<br>p.Arg276Ter	AD	Insertion/pathogenic	Pitt-Hopkins syndrome<br>OMIM: 610954
**17.**	F	DD, FD, muscular hypertonia, FTT	*CREBBP*	NM_004380.2<br>c.5641_5642delAG<br>p.Ser1881fs	AD	Frameshift/pathogenic	Rubinstein-Taybi syndrome 1<br>OMIM: 180849
**18.**	M	DD, hypotonia, involuntary movements, sensorineural deafness	*ALDH5A1*	NM_170740.1<br>c.1265G>A<br>p.Gly422Asp	AR	Missense/pathogenic	Succinic semialdehyde dehydrogenase deficiency<br>OMIM: 271980
**19.**	M	DD, FD, valvular pulmonary stenosis	*SOS1*	NM_005633.3<br>c.1367T>A<br>p.Phe623Ile	AD	Missense/likely pathogenic	Fibromatosis, gingival<br>OMIM: 135300
**20.**	F	DD, FD	*KMT2A*	NM_001197104.1<br>c.7789C>T<br>p.Gln2597X	AD	Insertion/likely pathogenic	Wiedemann-Steiner syndrome<br>OMIM: 605130

**21.**	F	DD, LA	*TMEM70*	NM_017866.6<br>c.317-2A>G	AD	Splicing/pathogenic	Mitochondrial complex V<br>(ATP synthase) deficien cy, nuclear type 2<br>OMIM: 614052
**22.**	F	ID, FD, hypotonia	*CAMK2A*	NM_001363989.1<br>C413C>T<br>p.Pro138Leu	AD	Missense/ unknown significance	Intellectual developmental disorder, autosomal dominant 53<br>OMIM: 617798
**23.**	F	ID	*MECP2*	NM_004992.3<br>c.916C>G<br>p.Arg306Cys	AD	Missense/pathogenic	PPM-X syndrome<br>OMIM: 300055
**24.**	M	DD, short stature, FD, congenital heart defect	*LZTR1*	NM_006767.4<br>c.850C>T<br>p.Arg284Cys	AD	Missense/pathogenic	Noonan syndrome 10<br>OMIM: 616564
**25.**	F	ID, FD, chronic renal insifficiency	*IFT140*	NM_014714.4<br>c.1565G>A<br>p.Gly522Glu	AD	Missense/likely pathogenic	Short-rib thoracic dysplasia 9 with or without polydactyly<br>OMIM: 266920
				NM_014714.4<br>c.874G>A<br>p.Val292Met		Missense/likely pathogenic	
**26.**	F	DD, short stature, FD	*RAF1*	NM_001354689.3<br>c.781C>T<br>p.Pro261Ser	AD	Missense/pathogenic	Noonan syndrome 5<br>OMIM: 611553
**27.**	M	DD, epilepsy	*SCL16A2*	NM_006517.5<br>c.45G>A<br>p.Trp15x	X	Insertion/likely pathogenic	Allan-Herndon-Dudley syndrome<br>OMIM: 300523

**28.**	F	DD, FD, microcephaly	*CHD3*	NM_001005271.3<br>c.5728C>G<br>p.Leu1910Val	AD	Missense/ unknown significance	Snijders Blok-Campeau syndrome<br>OMIM: 618205
			TUBB2B	NM_178012.5<br>c.1188C>G<br>p.His396Gln		Missense/ unknown significance	Cortical dysplasia, complex, with other brain malformations 7<br>OMIM: 610031
**29.**	F	DD, FD	*CHD3*	NM_001005271.3<br>c.2917C>T<br>p.Pro973Ser	AD	Missense/ unknown significance	Snijders Blok-Campeau syndrome<br>OMIM: 618205
**30.**	M	ID, epilepsy, psychomotor regression	*NPC1*	NM_000271.5<br>c.3182T>C<br>p.Ile1061Thr	AR	Missense/pathogenic	Niemann-Pick disease<br>OMIM: 257220
				NM_000271.5<br>c.2196dupT<br>p.Pro733fs		Frameshift/pathogenic	
**31.**	M	DD, microcephaly, seizures	*SPTAN1*	NM_001130438.2<br>c.6923_6928dupGC<br>ATGC<br>p.Arg2308_Met2309<br>dup	AD	Insertion/likely pathogenic	Developmental and epileptic encephalopathy 5<br>OMIM: 613477
**32.**	F	ID, SM	*UBE3A*<br>*EX9-10*<br>*DEL*	15q11.2<br>microdetetion<br>EX9-10 DEL	AD	Microdeletion/pathoge<br>nic	Angelman syndrome<br>OMIM: 105830
**33.**	F	DD, blindness	*PEX6*	NM_000287.4<br>c.1314_1321del<br>p.Leu438fs	AR	Frameshift/pathogenic	Peroxisome biogenesis disorder 4A (Zellweger)<br>OMIM: 614862

				NM_000287.4<br>c.160_243del<br>p.Val54_Leu81del		Deletion/pathogenic	
**34.**	M	DD, LA	*MT-ATP8*	NC_012920.1(ATP3_v001)<br>c.46A>C<br>p.Met16Leu	AR	Missense/ unknown significance	Mitochondrial compex V (ATP synthase) deficiency<br>OMIM: 516070
**35.**	M	DD	*STAG2, XIAP*	Xq25(122927376_123283576) x2	X	Microduplication/ pathogenic	Xq25 duplication syndrome OMIM: 300979
**36.**	F	DD	*ARHGEF9*	NM_001353923.1<br>c.601-2A>G	X	Splicing/likely pathogenic	Developmental and epileptic encephalopathy 8<br>OMIM:300607
**37.**	F	DD, epilepsy, microcephaly	*PCDH19*	NM_001105243<br>c.1081A>G<br>p.Ser361Gly	X	Nonsense/ likely pathogenic	Developmental and epileptic encephalopathy 9<br>OMIM:300088
**38.**	F	DD, renal dysplasia, coarctation of aorta, FD	*KMT2D*	NM_003482.3<br>c.10653_10654insT<br>p.Ala3552fs	AD	Frameshift/ likely pathogenic	Kabuki syndrome 1<br>OMIM:147920
**39.**	M	ID, macrocephaly, recurrent otitis media	*AGO2*	NM_012154.5<br>c.277G>A<br>p.Val93Met	AD	Missense/unknown significance	Lessel-Kreienkamp syndrome<br>OMIM:606229
**40.**	F	DD, movement disorder, recurrent otitis media, skin hypopigmentations	*ARID2*	NM_001347839<br>c.4181A>S<br>p.Glu1394Ala	AD	Missense/ unknown significance	Coffin-Siris syndrome 6<br>OMIM: 617808

**41.**	F	ID, short stature, FD	*GNAS*	NM_000516.4 c.296delT p.Leu99ArgfsTer12	AD	Frameshift/pathogenic	Pseudohypoparathyroidism Ia OMIM: 103580
**42.**	M	DD, epilepsy, LA	*PDHA1*	NM_000284.4 c.615C>G p.Phe205Leu	X	Missense/likely pathogenic	Pyruvate dehydrogenase E1-alpha deficiency OMIM:312170
**43.**	F	DD, hypotonia, FTT, LA	*PDHA1*	NM_001173454.1 c.597C>T p.(Tyr199=)	X	Missense/pathogenic	Pyruvate dehydrogenase E1-alpha deficiency OMIM:312170
**44.**	M	DD, hypotonia	*MED13L*	NM_015335.4 c.745A>T p.Lys249X	AD	Insertion/likely pathogenic	Impaired intellectual development and distinctive facial features with or without cardiac defects OMIM:616789
**45.**	F	DD, microcephaly, seizures	2q37 deletion sy (multiple genes)	arr(GRCh37)2q37.2 q37.3	AD	Microdeletion/pathogenic	Chromosome 2q37 deletion syndrome OMIM:600430
**46.**	F	ID, hypotonia, hyperactivity, ophthalmologic abnormalities, FD, hirsutism, cryptochidism	*SMARCC2*	NM_001330288.2 c.1833+1G>A	AD	Splicing/pathogenic	Coffin-Siris syndrome 8 OMIM:618362
**47.**	F	ID, hypotonia, movement disorder, sensorineural hearing impairment, bilateral ptosis, FD	*ATP8A2*	NM_016529.6 c.1058-2A>G	AR	Splicing/likely pathogenic	Cerebellar ataxia, impaired intellectual development, and dysequilibrium syndrome 4 OMIM:615268
**48.**	F	ID, developmental regression, hypotonia, optic nerve atrophy	*AIFM1*	NM_004208.4 c.1646C>T p.Ala549Val	X	Missense/ likely pathogenic	Spondyloepimetaphyseal dysplasia, X-linked,

							with hypomyelinating leukodystrophy OMIM:300232
**49.**	M	ID, hypotonia, cleft palate, club foot, feeding difficulties, growth delay, microcephaly, hearing impairment, crowded teeth, FD	*SATB2*	NM_001172509.2 c.847C>T p.Arg283Ter	AD	Nonsense/pathogenic	Glass syndrome OMIM:612313
**50.**	M	ID, attention deficit hyperactivity disorder, FD	*SETBP1*	NM_015559.3 c.1873C>T p.Arg625Ter	AD	Nonsense/pathogenic	Intellectual developmental disorder, autosomal dominant 29 OMIM:616078
**51.**	M	DD, truncal hypotonia, joint contractures, FTT, cryptorchidism	*SLC16A2*	NM_006517.5 c.611T>C	X	Missense/ likely pathogenic	Allan-Herndon-Dudley syndrome OMIM:300523
**52.**	F	DD, growth delay, microphthalmia, iris and choroidea colobomas, optic nerve atrophy, preauricular tags, hypospadia, mild perinatal asphyxia	*PUF60*	NM_078480.3 c.244-245del	AD	Frameshift/pathogenic	Verheij syndrome OMIM:615583
**53.**	M	DD, aplastic anemia, macrocephaly, FD, VSD, renal anomalies	*RPL5*	NM_000969.5 c.175_176delGA	AD	Frameshift/pathogenic	Diamond-Blackfan anemia 6 OMIM: 612561
**54.**	M	DD, VSD, WPW syndrome, FTT, myopia, strabismus, hyperactivity, FD	*ANKRD11*	NM_013275.6 c.3055A>G	AD	Missense/unknown significance	KBG syndrome OMIM: 148050
**55.**	F	DD, nystagmus, esotropia, hypotonia	*PLP1*	NM_000533.5 arr[GRCh37] Xq22.1q22.2(102240878_103104983)x2	X	Duplication/pathogenic	Pelizaeus-Merzbacher disease OMIM: 312080

**56.**	F	DD, hypotonia, cleft lip and palate, deafness, kyphoscoliosis, FD	*ACTG1*	NM_001614.5 c.359C>T p.Thr120Ile	AD	Missense/pathogenic	Baraitser-Winter syndrome 2 OMIM: 614583
**57.**	F	DD, autism, FD	*SHANK3*	NM_001372044.2 c.4365_4368del p.Asp1456ProfsTer 33	AD	Frameshift/pathogenic	Phelan-McDermid syndrome OMIM: 606232
**58.**	M	DD, hypotonia, involuntary movements, FD	*MECP2*	NM_001110792.2 c.509C>T p.Thr170Met	AD	Missense/pathogenic	Rett syndrome OMIM: 312750
**59.**	M	DD, hypotonia, convergent strabismus, difficulty swallowing	*CACNA1A*	NM_001127222.2 c.692T>G p.Leu231Arg	AD	Missense/ likely pathogenic	Developmental and epileptic encephalopathy 42 OMIM:617106
**60.**	F	DD, FTT, bilateral calcaneovalgus, FD, macrocephaly	*GCDH*	NM_000159.4 c.1063C>T p.Arg355Cys	AR	Missense/ likely pathogenic	Glutarica ciduria, type I OMIM: 231670
				NM_000159.4 c.1169G>C p.Gly390Ala		Missense/pathogenic	
**61.**	F	DD, severe hypotonia, FD, agenesis of the corpus callosum, trigonocephaly, tracheoesophageal fistula, coarctation of the aorta, persistent ductus arteriosus, VSD, micropenis, cryptorchidism, umbilical hernia, hydronephrosis, talipes equinovarus, camptodactyly, long thumbs, pale skin and hypothyreosis	*KAT6B*	NM_012330.4 c.3670dupA p.Met1224AsnfsTer 3	AD	Frameshift/pathogenic	SBBYSS syndrome OMIM:603736
**62.**	M	DD, epilepsy, tremor,behavior problems, ischemic brain lesions	*CNOT3*	NM_003482.4 c.926A>C	AD	Missense/unknown significance	Intellectual developmental disorder

				p.His309Pro			with speech delay, autism, and dysmorphic facies<br>OMIM: 618672
**63.**	F	Microphthalmia, chorioretinal coloboma, anal atresia, cleft palate, gastroesophageal reflux, FTT, FD, hypotonia, DD	*KMT2D*	NM_003482.4<br>c.13575dupA<br>p.Lys4527GlufsTer48	AD	Frameshift/pathogenic	Kabuki syndrome 1<br>OMIM: 147920
**64.**	M	DD, epilepsy	*KCNQ2*	NM_172107.4<br>c.901G>A<br>p.Gly301Ser	AD	Missense/pathogenic	Developmental and epileptic encephalopathy 7<br>OMIM: 613720
**65.**	F	DD, hypotonia, hearing loss, FD, microcephaly, ASD, FTT	*AFF4*	NM_014423.4<br>c.772C>T<br>p.Arg258Trp	AD	Missense/ likely pathogenic	CHOPS syndrome<br>OMIM: 616368
**66.**	M	DD, congenital brain anomalies, microcephaly, epilepsy, growth retardation, feeding problems, FD	*BPTF*	NM_182641.4<br>c.4416G>A<br>p.Met1472Ile	AD	Missense/unknown significance	Neurodevelopmental disorder with dysmorphic facies and distal limb anomalies<br>OMIM: 617755

Legend: DD – developmental delay, ID – intellectual disability, FD – facial dysmorphism, SD – skeletal dysplasia, LA– lactic acidosis, SM – stereotypic movements, FTT – failure to thrive, VSD – ventricular septal defect
